# Outcome of Patients with Pregnancy-Associated Breast Cancer Who Have Subsequent Pregnancies

**DOI:** 10.1245/s10434-024-15798-5

**Published:** 2024-07-10

**Authors:** Alissa Doll, Marla Lipsyc-Sharf, Myung Shin Sim, Jennifer L. Baker, Nimmi S. Kapoor

**Affiliations:** 1 Department of Surgery, Division of Surgical Oncology, David Geffen School of Medicine, University of California Los Angeles, Los Angeles, CA USA; 2 Department of Medicine, Division of Hematology-Oncology, David Geffen School of Medicine, University of California Los Angeles, Los Angeles, CA USA; 3 Division of General Internal Medicine and Health Services Research, University of California Los Angeles, Los Angeles, CA USA

## Abstract

**Background:**

After treatment of pregnancy-associated breast cancer (PABC), some women desire future pregnancy. While safety of pregnancy after breast cancer has been demonstrated, the same cannot be said about women with PABC.

**Objective:**

The aim of this study was to describe the incidence and outcomes of patients with PABC with subsequent pregnancies compared with those without another pregnancy.

**Methods:**

A retrospective chart review identified patients diagnosed with breast cancer during pregnancy or within 5 years postpartum between 2011 and 2023. Patients were then screened for further pregnancy. Clinicopathologic variables, oncologic outcomes, and pregnancy outcomes were recorded. The Chi-square test and t-test were used to compare patients with subsequent pregnancy with those without. Kaplan–Meier method and log-rank test were used to estimate 5-year disease-free survival (DFS).

**Results:**

Overall, 75 patients with PABC were identified, 58 of whom had PABC and no further pregnancies (NSP-PABC) and 17 with subsequent pregnancy (SP-PABC). Compared with patients with NSP-PABC, patients with SP-PABC were significantly younger (*p* = 0.015) and less likely to have prior pregnancies (*p* < 0.001). Overall median follow-up was 4.3 years. Calculated 5-year DFS rates were 86.2% and 89.0% for the SP-PABC and NSP-PABC groups, respectively (*p* = 0.76). Calculated 5-year overall survival was 100% and 90.7% for the SP-PABC and NSP-PABC groups, respectively (*p* = 0.22). Within the SP-PABC group, 14/17 patients had successful deliveries.

**Conclusions:**

This study provides the first descriptions of patients with PABC and subsequent pregnancy. Additional investigation, likely with pooled analysis from multiple institutions, is necessary to determine the oncologic and obstetric safety of pregnancy following PABC.

Women diagnosed with breast cancer during or shortly after pregnancy endure unique diagnostic and treatment challenges. Special considerations regarding both locoregional and systemic therapy are tailored to the unique circumstance of the cancer and the timing of the cancer diagnosis with respect to pregnancy.^1,2^ As circumstantial and biological understanding of pregnancy-associated breast cancer (PABC) has grown, the definition of PABC has evolved. Historically, PABC has been defined by its association to pregnancy, lactation, and the post-partum period, most commonly within the first-year post-partum.^3,4^ More recently however, the definition of PABC has expanded to include patients diagnosed with breast cancer up to 5 years post-partum due to increased understanding of the long-term hormonal effects from pregnancy on breast tissue.^5,6^ It is important to distinguish PABC as such since PABC is considered a biologically and prognostically distinct entity compared with breast cancer not associated with pregnancy.^7^ At the same time, it is also understood that patients who develop breast cancer while pregnant have a more favorable prognosis than patients who develop breast cancer in the post-partum period.^8,9^

Nonetheless, regardless of timing of pregnancy and breast cancer, patients with PABC may desire future pregnancy. In fact, as the maternal age rises and the incidence of breast cancer diagnosed in young women (<40 years of age) increases,^10,11^ increasing numbers of young women consider their families to be incomplete after treatment of breast cancer.^12^ The desire for fertility preservation has led to an increased focus on oncofertility counseling and treatment as an integral component of breast cancer treatment in premenopausal women.^13,14^ While oncologic safety of pregnancy after breast cancer, in general, has been demonstrated in the POSITIVE trial^15^ as well as various observational meta-analyses,^16,17^ the same cannot be said about subsequent pregnancy specifically in women with PABC. The clinical course and safety of subsequent pregnancy following treatment for PABC is a subject that has not been thoroughly explored and is not well understood.

Therefore, in this study, we aimed to describe the incidence and clinical outcomes of patients with PABC who have subsequent pregnancies after their breast cancer treatment compared with those with PABC who do not have subsequent pregnancies.

## Methods

A single-institution, observational, retrospective chart review, determined to meet criteria for exemption by our Institutional Review Board, identified consecutive patients diagnosed with PABC between 2010 and 2023 who were treated at our institution. PABC was defined as a breast cancer diagnosed during pregnancy or within 5 years postpartum. Records of patients with PABC were then screened for any further pregnancy. Subsequent pregnancy after PABC (SP-PABC) defined patients who had a documented pregnancy any time after their breast cancer diagnosis during their follow-up, and no subsequent pregnancy after PABC (NSP-PABC) defined patients who did not have a documented pregnancy after their breast cancer diagnosis within their follow-up.

Patient characteristics included pregnancy history prior to breast cancer diagnosis, fertility treatment prior to breast cancer diagnosis, genetic predisposition, and first-degree family members with a history of breast cancer. Tumor characteristics included grade, tumor subtype, T stage, N stage, M stage, overall stage, and receptor status. Treatment course included receipt of neoadjuvant chemotherapy, type of breast surgery, type of axillary surgery, reconstruction, type of reconstruction, adjuvant radiation, adjuvant chemotherapy, duration of endocrine therapy, and ovarian suppression at any point during their breast cancer treatment.

For patients who presented with stage 0–III breast cancer, disease-free survival (DFS), distant DFS (DDFS), and overall survival (OS) were evaluated. For patients within the SP-PABC group, the details and outcome of subsequent pregnancies, including timing of subsequent pregnancy from the date of breast cancer diagnosis, age at subsequent pregnancy, outcome of pregnancy (i.e. successful birth), and gestational age at the time of delivery, were recorded.

The Chi-square test for categorical variables and two-sample t-test for continuous variables were used to compare SP-PABC with NSP-PABC. Two-sided tests with a *p*-value <0.05 indicated a significant difference. The Kaplan–Meier method and log-rank test were used to estimate 5-year DFS and OS. Analyses were performed using SAS 9.4 (SAS Institute, Inc., Cary, NC, USA).

## Results

### Study Characteristics

Seventy-five patients with PABC were identified during the study period, 58 of whom had PABC and no further pregnancies (NSP-PABC) and 17 of whom had documented subsequent pregnancy (SP-PABC) [Table 1]. The median age of the study population at breast cancer diagnosis was 36 years (range 25–41 years). Overall, approximately one-third of patients were diagnosed with PABC during pregnancy and two-thirds were diagnosed post-partum. Among all patients with PABC diagnosed with breast cancer during pregnancy (*n* = 25), 12 were diagnosed during their first trimester (48%), 9 were diagnosed during their second trimester (36%), and 4 were diagnosed during their third trimester (16%). Among all patients with PABC diagnosed post-partum (*n* = 50), the median time from delivery to breast cancer diagnosis was 22 months (range 2–54 months).

**Table 1:** Patient characteristics and breast cancer treatment

	SP-PABC [*n* = 17]	NSP–PABC [*n* = 58]	Total [*n* = 75]	*p*-Value
Timing of pregnancy and breast cancer diagnosis				
Gestational Postpartum	4 (23.5) 13 (76.5)	21 (36.2) 37 (63.8)	25 (33.3) 50 (66.7)	0.330
Age at breast cancer diagnosis, years (mean ± SD)	32.9 ± 4.1	35.7 ± 4.1	35.1 ± 4.2	*0.015*
Race				
White Black Asian Other	9 (52.9) 2 (11.8) 2 (11.8) 4 (23.5)	26 (44.8) 7 (12.1) 10 (17.2) 15 (25.9)	35 (46.7) 9 (12) 12 (16) 19 (25.3)	0.927
Genetic predisposition				
Yes No Unknown	4 (23.5) 11 (64.7) 2 (11.8)	13 (22.4) 42 (72.4) 3 (5.2)	17 (22.7) 53 (70.7) 5 (6.7)	0.614
Prior pregnancy before PABC				
Yes No	2 (11.8) 15 (88.2)	35 (60.3) 23 (39.7)	37 (49.3) 38 (50.7)	*< 0.001*
Fertility treatment				
Yes No Unknown	1 (5.9) 15 (88.2) 1 (5.9)	3 (5.2) 46 (79.3) 9 (15.5)	4 (5.3) 61 (81.3) 10 (13.3)	0.590
First-degree relative with breast cancer				
Yes No Unknown	5 (29.4) 12 (70.6) 0 (0)	9 (15.5) 47 (81.0) 2 (3.5)	14 (18.7) 59 (78.7) 2 (2.7)	0.345
T stage at diagnosis				
0 1 2 3 4 Unknown	4 (23.5) 3 (17.7) 8 (47.1) 0 (0) 1 (5.9) 1 (5.9)	4 (6.9) 17 (29.3) 28 (48.3) 7 (12.1) 2 (3.5) 0 (0)	8 (10.7) 20 (26.7) 36 (48.0) 7 (9.3) 3 (4.0) 1 (1.3)	0.083
N stage at diagnosis				
0 1 2 3 Unknown	11 (64.7) 4 (23.5) 1 (5.9) 0 (0) 1 (5.9)	34 (58.6) 15 (25.9) 5 (8.6) 4 (6.9) 0 (0)	45 (60) 19 (25.3) 6 (8.0) 4 (5.3) 1 (1.3)	0.307
Overall stage at diagnosis				
0 I II III IV	4 (23.5) 3 (17.7) 7 (41.2) 3 (17.7) 0 (0)	4 (6.9) 14 (24.1) 24 (41.4) 10 (17.2) 6 (10.3)	8 (10.7) 17 (22.7) 31 (41.3) 13 (17.3) 6 (8.0)	0.247
Tumor subtype				
ER+/HER2− ER+/HER2+ ER−/HER2+ ER−/HER2− ER+ (DCIS) ER− (DCIS) Unknown	4 (23.5) 1 (5.9) 2 (11.8) 6 (35.3) 2 (11.8) 1 (5.9) 1 (5.9)	20 (34.5) 12 (20.7) 4 (6.9) 18 (31.0) 3 (5.2) 1 (1.7) 0 (0)	24 (32) 13 (17.3) 6 (8.0) 24 (32.0) 5 (6.7) 2 (2.6) 1 (1.3)	0.258
Neoadjuvant chemotherapy				
Yes No	8 (47.1) 9 (52.9)	34 (58.6) 24 (41.4)	42 (56.0) 33 (44.0)	0.398
Adjuvant chemotherapy				
Yes No Unknown	5 (29.4) 12 (70.6) 0 (0)	32 (55.2) 25 (43.1) 1 (1.7)	37 (49.3) 37 (49.3) 1 (1.3)	0.130
Breast surgery				
Lumpectomy Unilateral mastectomy Bilateral mastectomy None	5 (29.4) 1 (5.9) 11 (64.7) 0 (0)	16 (27.6) 12 (20.7) 27 (46.6) 3 (5.2)	21 (28.0) 13 (17.3) 38 (50.7) 3 (4.0)	0.332
Axillary surgery				
None SLNB ALND Unknown	0 (0) 13 (76.5) 3 (17.7) 1 (5.9)	4 (6.9) 43 (74.1) 11 (19.0) 0 (0)	4 (5.3) 56 (74.7) 14 (18.7) 1 (1.3)	0.203
Reconstruction				
Yes No NA (lump, no surgery)	12 (70.6) 0 (0) 5 (29.4)	34 (58.6) 5 (8.6) 19 (32.8)	46 (61.3) 5 (6.7) 24 (32.0)	0.403
Radiation therapy				
Yes No	9 (52.9) 8 (47.1)	30 (51.7) 28 (48.3)	39 (52) 36 (48.0)	0.929
Oral endocrine therapy				
Yes No	5 (29.4) 12 (70.6)	26 (44.8) 32 (55.2)	31 (41.3) 44 (58.7)	0.256
Duration of endocrine therapy, months [mean ± SD]	59.8 ± 43.7	41.4 ± 28.5	44.3 ± 31.0	0.425
Ovarian suppression				
Yes No	2 (11.8) 15 (88.2)	16 (27.6) 42 (72.4)	18 (24) 57 (76)	0.179

Data are expressed as *n* (%) unless otherwise specified

*SP-PABC* subsequent pregnancy after pregnancy-associated breast cancer, *NSP-PABC* no subsequent pregnancy after pregnancy-associated breast cancer, *PABC* pregnancy-associated breast cancer, *ER* estrogen receptor, *HER2* human epidermal growth factor receptor 2, *DCIS* ductal carcinoma in situ, *SLNB* sentinel lymph node biopsy, *ALND* axillary lymph node dissection, *SD* standard deviation, *NA* not available

Overall, 17 (22.7%) patients had a known pathogenic genetic mutation, including six (35.3%) patients with BRCA1 mutations, three (17.6%) with CHEK2 mutations, two (11.8%) with BRCA2 mutations, two (11.8%) with ATM mutations, one patient each with a mutation in MSH2, NBN, or APC, and one patient with multiple mutations (BRCA1, ATM, and MUTYH). Among all patients, only 14 (18.7%) had a first-degree family member previously diagnosed with breast cancer. Most commonly, patients had stage II disease (*n* = 31, 41.3%) at the time of diagnosis and six patients (8.0%) had stage IV disease at the time of diagnosis. Approximately half (*n* = 37, 49.3%) of all patients had invasive estrogen receptor-positive (ER+) breast cancers, approximately one-third had invasive triple-negative breast cancers, with the remaining patients having ER-negative (ER−), human epidermal growth factor receptor 2-positive (HER2+) invasive breast cancers or ductal carcinoma in situ (DCIS).

With regard to treatment, 56% of patients received neoadjuvant chemotherapy. Approximately half of the patients underwent bilateral mastectomy (50.7%) and the most commonly performed axillary surgery was sentinel lymph node biopsy (74.7%). The majority of patients who underwent either bilateral or unilateral mastectomy underwent subsequent reconstruction (90.2%), with the most common reconstruction being tissue expander/implant-based.

Compared with patients with NSP-PABC, patients with SP-PABC were significantly younger (mean age 32 years vs. 36 years; *p* = 0.015) and were less likely to have had any pregnancy prior to that associated with their breast cancer diagnosis (*p* < 0.001) [Table 1]. Otherwise, patients in both groups had similar baseline characteristics, including the timing of breast cancer diagnosis in relation to pregnancy, presence of a genetic predisposition, first-degree family members with breast cancer, and prior fertility treatments. The two groups exhibited similar tumor characteristics including T stage, N stage, overall stage, and tumor subtype. Six patients with de novo metastatic disease were identified, all of whom were within the NSP-PABC group. These patients were noted in raw data description but were not included in DFS, DDFS, or OS analyses. Locoregional and systemic cancer treatments, including receipt of neoadjuvant chemotherapy, type of breast surgery, type of axillary surgery, reconstruction, receipt of adjuvant chemotherapy, receipt of irradiation, duration of endocrine therapy, and receipt of ovarian suppression, were also similar between both patient groups.

### Survival

**Fig. 1:**
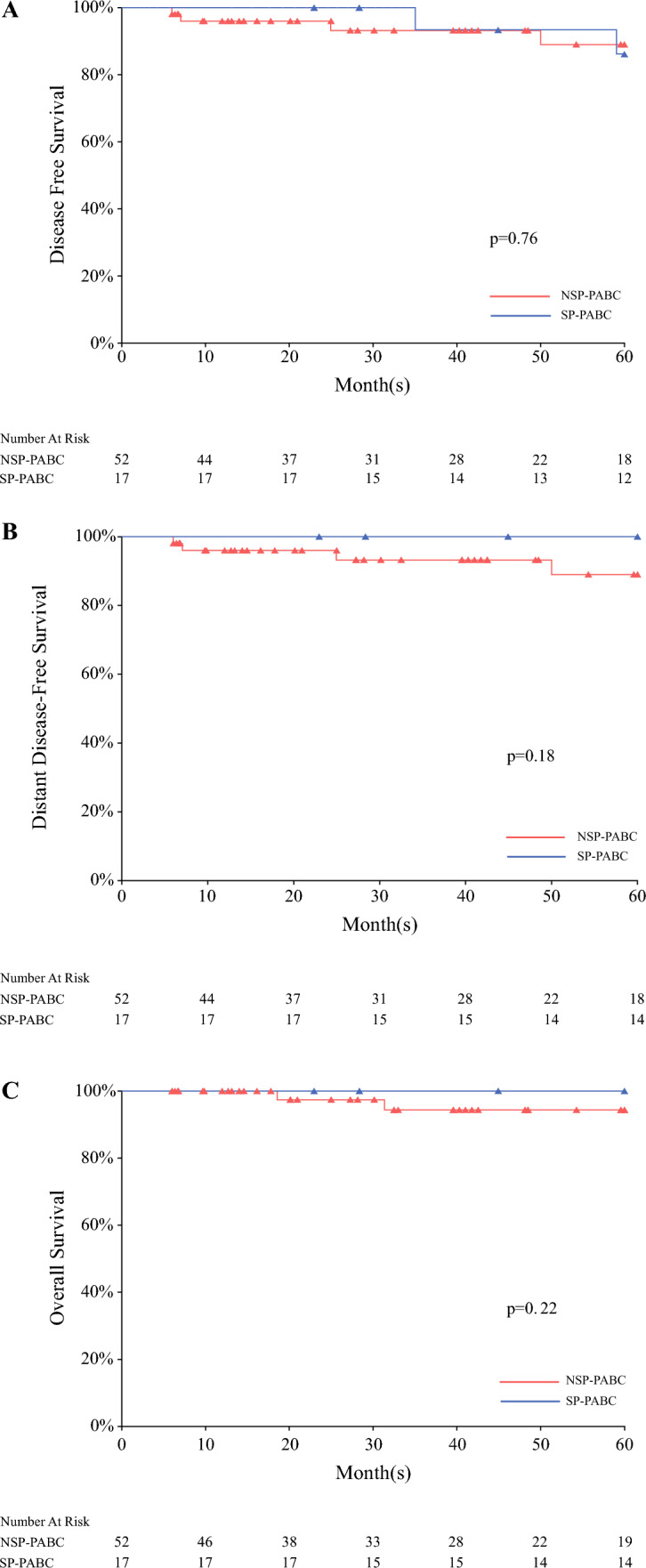
5-year Kaplan-Meier Survival Curves. **A** Disease-free survival. **B** Distant disease-free survival. **C** Overall survival. NSP-PABC—no subsequent pregnancy after pregnancy associated breast cancer. SP-PABC—subsequent pregnancy after pregnancy associated breast cancer.

Overall median follow-up time was 4.3 years (range 1.0–11.8 years), with a median of 7.3 years in the SP-PABC group and 3.3 years in the NSP-PABC group. Of patients who presented with non-metastatic disease, calculated 5-year DFS rates were similar at 86.2% and 89.0% for the SP-PABC and NSP-PABC groups, respectively (*p* = 0.76). Calculated 5-year OS was 100% in the SP-PABC group and 90.7% in the NSP-PABC group (*p* = 0.22). Freedom from distant metastasis at 5 years was 100% in the SP-PABC group and 89.0% in the NSP-PABC group (*p* = 0.18) (Fig. 1).

### Subsequent Pregnancy After Pregnancy-Associated Breast *Cancer*

Of the 17 patients who had an SP-PABC, three patients were diagnosed with breast cancer during the first trimester (17.6%), one was diagnosed during the second trimester (5.9%), and 13 were diagnosed post-partum (76.5%), with a median time from delivery to breast cancer diagnosis of 30 months (range 8–54 months) for the patients with postpartum breast cancer (Table 2). Sixteen of 17 patients with PABC carried the pregnancy to delivery and one underwent a therapeutic abortion.

**Table 2:** Characteristics of patients with subsequent pregnancy after pregnancy-associated breast cancer

Patient	Age at PABC Dx, years	Genetic mutation	Relation of pregnancy to cancer diagnosis	PABC pregnancy outcome	Tumor type	Biomarkers	Stage	Systemic treatment	Surgical treatment	Time from PABC to next pregnancy (months)	Outcome of subsequent pregnancy	Locoregional or distant cancer recurrence	Follow-up (years)	Death
1	31	None	First trimester	TAB	Unknown	ER+/HER2+	Unknown	Neoadjuvant ACTH/TAM	PM/NA	42	SAB	No	9	No
2	31	None	14 months PP	Delivery	IDC	ER+/HER2−	T1N0	None/TAM	BM/SLNB	55	Term infant	No	11.2	No
3	41	None	First trimester	Delivery	DCIS	ER+	Tis	None	BM/SLNB	16	Term infant	No	2	No
4	36	CHEK2	32 months PP	Delivery	DCIS	Not tested	Tis	None	BM/SLNB	23	Trisomy 13	No	5.4	No
5	36	BRCA1	8 months PP	Delivery	IDC	ER+/HER2−	T2N1	Adjuvant TC/AI	BM/SLNB	34	Pre-term infant	Yes – axillary	4.3	No
6	27	None	25 months PP	Delivery	IDC	ER−/HER2+	T2N2	TCH	BM/ALND	19	Term infant	No	11.8	No
7	32	No test	First trimester	Delivery	DCIS	ER−	Tis	None	PM/SLNB	23	Term infant	No	4.3	No
8	32	None	48 months PP	Delivery	IDC	ER−/HER2−	T4dN1	TC	BM/ALND	16	Term infant	No	7.75	No
9	33	No test	22 months PP	Delivery	IDC	ER−/HER2−	T2N0	TC	L/SLNB	25	Term infant	No	8.8	No
10	35	None	36 months PP	Delivery	IDC	ER−/HER2+	T2N1	TCHP	BM/ALND	87	Term infant	No	8.6	No
11	41	BARD1	36 months PP	Delivery	DCIS	ER+	Tis	None	BM/SLNB	53	SAB	Yes – axillary	7.5	No
12	32	None	54 months PP	Delivery	IDC	ER+/HER2−	T1N0	TC	L/SLNB	22	Unknown	No	2.3	No
13	33	None	24 months PP	Delivery	IDC	ER+/HER2−	T2N1	TC	UM/SLNB	74	Term infant	No	7.3	No
14	30	None	Second trimester	Delivery	IDC	ER−/HER2−	T1N0	TAM	L/SLNB	42	Term infant	No	6.6	No
15	25	None	54 months PP	Delivery	IDC	ER−/HER2−	T2N0	TAC	BM/SLNB	36	Term infant	No	9/5	No
16	32	None	30 months PP	Delivery	IDC	ER−/HER2−	T2N0	TC	BM/SLNB	24	Term infant	No	5.7	No
17	32	PALB2	22 months PP	Delivery	IDC	ER−/HER2−	T1N0	TAC	BM/SLNB	59	Term infant	No	6.8	No

*PABC* pregnancy-associated breast cancer, *Dx* diagnosis, *PP* post-partum, *TAB* therapeutic abortion, *ER* estrogen receptor, *HER2* human epidermal growth factor receptor 2, *ACTH* doxorubicin-cyclophosphamide-paclitaxel-trastuzamab, *TC* taxotere-cyclophosphamide, *TCH* taxotere-carboplatin-herceptin, *TCHP* taxotere-carboplatin-herceptin-pertuzumab, *TAC* taxotere-adriamycin-cyclophosphamide, *TAM* tamoxifen, *AI* aromatase inhibitor, *PM* partial mastectomy, *BM* bilateral mastectomy, *SLNB* sentinel lymph node biopsy, *ALND* axillary lymph node dissection, *SAB* spontaneous abortion, *IDC* invasive ductal carcinoma, *DCIS* ductal carcinoma in situ

Within the SP-PABC group, all patients with invasive cancer received some form of systemic therapy (chemotherapy, endocrine therapy, or both). Five patients (29.4%) were receiving endocrine therapy, with a median time of 50.5 months (range 18–120 months). Three patients discontinued endocrine therapy at 18, 30, and 41 months. Only the one patient who discontinued at 18 months did so to attempt pregnancy, whereas reasons for discontinuation in the remaining two patients were not clearly related to desired pregnancy. The remaining 12 patients were not treated with endocrine therapy, including eight patients who had ER− tumors, three with ER+ DCIS who underwent bilateral mastectomy, and one who declined endocrine therapy.

At a mean follow-up of 7.3 years, 2/17 patients (11.8%) with SP-PABC developed locoregional recurrence within the axilla, with no other evidence of disease, and both were treated with axillary lymph node dissection. Neither patient developed distant disease at 5.25 and 7.5 years from their initial breast cancer diagnosis (2.25 and 4.5 years from axillary recurrence, respectively). One patient who developed an axillary recurrence experienced spontaneous abortion in her subsequent pregnancy. The second patient who developed an axillary recurrence was BRCA1-positive and was diagnosed with her axillary recurrence during her subsequent pregnancy. Importantly, there were no observed distant recurrences or deaths in the SP-PABC patients. Within the SP-PABC group, timing from PABC diagnosis to subsequent pregnancy was an average of 34 months (range 16–87 months) and at least two patients underwent in vitro fertility for their subsequent pregnancies. 14/17 (82.4%) patients had live births, including 12 (85.7%) full-term infants, one (7.1%) pre-term infant, and one infant (7.1%) diagnosed with trisomy 13 who died shortly after birth.

### No Subsequent Pregnancy After Pregnancy-Associated Breast Cancer

Within the NSP-PABC group, breast cancer was diagnosed during the first trimester in nine patients (15.5%), second trimester in eight patients (13.8%), and third trimester in four patients (6.9%). Thirty-seven women (63.8%) were diagnosed with breast cancer within 5 years post-partum, with a median time from delivery to breast cancer diagnosis of 18.5 months (range 2–52 months). Of the 58 patients with NSP-PABC, one developed locoregional recurrence (1.7%), four developed distant recurrence (6.9%), and there were six deaths due to breast cancer (10.3%). The patient who developed a local recurrence was treated with mastectomy and repeat axillary staging via sentinel lymph node biopsy. This patient had no evidence of distant disease at recent follow-up, which was 10 years from the initial diagnosis (3 years from diagnosis of recurrence). Of the four patients within the NSP-PABC group who developed distant recurrence, three suffered a breast cancer-related death. Of the six patients who presented with metastatic disease, 50% remain living during the study follow-up period (median 6.5 years, range 2.17–6.5 years).

## Discussion

PABC and breast cancer in young women in general are each increasing in frequency.^10,11^ Simultaneously, as the maternal age rises, increasing numbers of women are diagnosed with breast cancer prior to completion of their families.^18^ This study provides the first descriptions of patients with PABC and subsequent pregnancy. Women pursuing pregnancy following PABC tended to be younger and were less likely to have children prior to breast cancer diagnosis compared with those who did not pursue additional pregnancy. While we did not observe statistically significant differences between the SP-PABC and NSP-PABC groups, relatively fewer patients in the SP-PABC group underwent any systemic chemotherapy, and more patients in the SP-PABC group presented with stage 0 breast cancer (Table 1). Although our cohort is small, there did not appear to be increased risk of a distant recurrence or breast cancer-related mortality in patients with SP-PABC compared with patients with PABC who did not have subsequent pregnancies. Furthermore, extended median follow-up time of 7.25 years within the SP-PABC group, compared with 3.3 years in the NSP-PABC group, provides additional reassurance of these outcomes.

Numerous studies have demonstrated the oncologic safety of pregnancy following breast cancer treatment. In the POSITIVE trial, patients with stage I–III hormone receptor-positive breast cancer who desired pregnancy interrupted endocrine therapy after 18–30 months of treatment. Of 497 women who were followed, 368 (74.0%) became pregnant and 317 (63.8%) had at least one live birth. At a median follow-up of 41 months, there were 44 (8.9%) total breast cancer events in patients who interrupted endocrine therapy and 168 (9.2%) in the control cohort. This study concluded that patients who became pregnant did not have an increased risk of breast cancer-related events.^15^ Similarly, in our study, which in contrast only included patients with breast cancer diagnosed during or shortly after pregnancy, there was not an increased risk of recurrence or breast cancer-related mortality in women with subsequent pregnancy compared with those without.

Considering oncologic outcomes and pregnancy further, it is known that patients who develop post-partum breast cancer have worse prognosis than those who develop breast cancer while pregnant.^8^ A 2016 meta-analysis of 41 studies compared OS and DFS in patients diagnosed with breast cancer during pregnancy or within 5 years postpartum compared with controls with breast cancer and no pregnancy^.^^9^ Women diagnosed with breast cancer in the postpartum period had the worst OS (hazard ratio [HR] 1.79, 95% confidence interval [CI] 1.39–2.29) and DFS (HR 1.51, 95% CI 1.22–1.88) compared with controls. In our study, one-third of patients (*n* = 25) had gestational breast cancer, while two-thirds (*n* = 50) had post-partum breast cancer, and this was similar between the SP-PABC and NSP-PABC groups (Table 1). Larger studies with longer follow-up will be useful to see if there is a difference in outcomes among these groups in relation to timing of pregnancy, breast cancer, and subsequent pregnancy.

The oncologic safety of pregnancy following breast cancer treatment has been further reinforced by a large meta-analysis that included over 7500 women who became pregnant after their breast cancer diagnosis.^17^ Many of these pregnancies ultimately resulted in live births. However, compared with the general population, there was both an increased risk of cesarean section for these women, as well as an increased risk of prematurity, low birth weight, and small for gestational age for babies born to these women treated for breast cancer.^17^ In a separate meta-analysis of 16 studies, the rates of pregnancy among patients with a prior history of breast cancer were, on average, 40% lower compared with the general population, and among those who did become pregnant, the average rate of pregnancy loss was 12%.^19^ Our study exhibited a similar rate of pregnancy loss following PABC treatment (11.8%); however, details about method of delivery and birth weight were not captured in our study since many women delivered outside of our institution. Nonetheless, additional counseling and psychosocial support are needed for this population, with a focus on these challenges.

Genetic predisposition also needs to be considered as young women with breast cancer are more often found to have genetic risk compared with older patients. In our study, 22.7% of women carried a pathogenic mutation, with similar rates in both the SP-PABC and NSP-PABC groups. In 2024, Lambertini et al. published a retrospective cohort study investigating outcomes specifically in germline BRCA-positive patients who pursued pregnancy following breast cancer diagnosis. This study included 4732 patients, 659 of whom pursued pregnancy following breast cancer diagnosis. Overall, BRCA-positive patients who became pregnant did not have worsened DFS but instead exhibited improved OS compared with BRCA-positive patients who did not become pregnant.^20^ Although published data from both the POSITIVE trial and the recent Lambertini et al. study do not specify a subgroup of patients with PABC, it is possible that some of the patients within these large cohorts of patients had PABC.

Most studies of breast cancer outcomes and pregnancy after breast cancer tend to exclude women with stage IV breast cancer,^15,20^ as women with metastatic breast cancer are unlikely to pursue pregnancy. Our data collection yielded patients diagnosed with stage IV PABC that does warrant descriptive analysis, as given in Table 1; however, to align with previous studies and to more equally compare groups, we excluded patients with stage IV breast cancer in our survival analyses.

### Limitations

Retrospective studies related to pregnancy are limited by the ability to identify attempted pregnancy and potentially unreported miscarriages or early spontaneous abortions that occur early in pregnancy and are not captured in standard medical record charts. Likewise, completeness of records and data on subsequent pregnancies, including detail on the use of fertility treatment, rates of cesarean section, and birth weights, were not available. In addition, while median follow-up was 4.3 years in our study, some patients had only 1-year follow-up. It is plausible that with longer follow-up, more patients in the NSP-PABC group may also have subsequent pregnancy. There may also be potential for selection bias among patients who choose to undergo subsequent pregnancy due to oncologic or patient factors. Furthermore, this was a small study and although our two cohorts did not show statistically significant differences, conclusions about long-term outcome and survival are limited and larger studies with longer follow-up are warranted. Nonetheless, it is the first study to describe a group of patients with PABC and subsequent pregnancy and provide some comparison with those patients with PABC and no further reported pregnancy.

## Conclusion

In our study, we observed a cohort of patients with pregnancy after PABC and found a low breast cancer recurrence rate and high rates of subsequent pregnancy resulting in live births within this group. Younger patients with PABC who have fewer children are more likely to desire additional pregnancy, and our small series provides initial data supporting that this may be a safe endeavor. While these early data are promising, additional investigation, likely with pooled analyses from multiple institutions, is necessary to determine the oncologic and obstetric safety of pregnancy following PABC.

## References

[1] National Comprehensive Cancer Network. NCCN Clinical Practice Guidelines in Oncology (NCCN Guidelines). Breast Cancer. Accessed April 16, 2024.

[2] Loibl S, Azim HA, Bachelot T, et al. ESMO expert consensus statements on the management of breast cancer during pregnancy (PrBC). *Annals of Oncology*. 2023;34(10):849–66. 10.1016/j.annonc.2023.08.001. 37572987 10.1016/j.annonc.2023.08.001

[3] Johansson ALV, Stensheim H. Epidemiology of pregnancy-associated breast cancer. *Adv Exp Med Biol*. 2020;1252:75–9. 10.1007/978-3-030-41596-9_9. 32816264 10.1007/978-3-030-41596-9_9

[4] Gooch JC, Chun J, Kaplowitz E, Guth A, Axelrod D, Shapiro R, Roses D, Schnabel F. Pregnancy-associated breast cancer in a contemporary cohort of newly diagnosed women. *Breast J*. 2020;26(4):668–71. 10.1111/tbj.13510. 31448522 10.1111/tbj.13510

[5] Iqbal J, Amir E, Rochon PA, Giannakeas V, Sun P, Narod SA. Association of the timing of pregnancy with survival in women with breast cancer. *JAMA Oncol*. 2017;3(5):659–65. 10.1001/jamaoncol.2017.0248. 28278319 10.1001/jamaoncol.2017.0248 PMC5824205

[6] Proussaloglou EM, Blanco LZ Jr, Siziopikou KP. Updates in the pathology of Pregnancy Associated Breast Cancer (PABC). *Pathol Res Pract*. 2023;244:154413. 10.1016/j.prp.2023.154413. 36921545 10.1016/j.prp.2023.154413

[7] Lee GE, Mayer EL, Partridge A. Prognosis of pregnancy-associated breast cancer. *Breast Cancer Res Treat*. 2017;163(3):417–21. 10.1007/s10549-017-4224-6. 28365832 10.1007/s10549-017-4224-6

[8] Amant F, Lefrère H, Borges VF, et al. The definition of pregnancy-associated breast cancer is outdated and should no longer be used. *Lancet Oncol*. 2021;22(6):753–4. 10.1016/S1470-2045(21)00183-2. 34087122 10.1016/S1470-2045(21)00183-2 PMC8868503

[9] Hartman EK, Eslick GD. The prognosis of women diagnosed with breast cancer before, during and after pregnancy: a meta-analysis. *Breast Cancer Res Treat*. 2016;160(2):347–60. 10.1007/s10549-016-3989-3. 27683280 10.1007/s10549-016-3989-3

[10] Tesch ME, Partridge AH. Treatment of breast cancer in young adults. *Am Soc Clin Oncol Educ Book*. 2022;42:1–12. 10.1200/EDBK_360970. 35580291 10.1200/EDBK_360970

[11] Xu S, Murtagh S, Han Y, Wan F, Toriola AT. Breast cancer incidence among US women aged 20 to 49 years by race, stage, and hormone receptor status. *JAMA Netw Open*. 2024;7(1):e2353331. 10.1001/jamanetworkopen.2023.53331. 38277147 10.1001/jamanetworkopen.2023.53331 PMC10818222

[12] Bell RJ, Fradkin P, Parathithasan N, Robinson PJ, Schwarz M, Davis SR. Pregnancy-associated breast cancer and pregnancy following treatment for breast cancer, in a cohort of women from Victoria, Australia, with a first diagnosis of invasive breast cancer. *Breast*. 2013;22(5):980–5. 10.1016/j.breast.2013.05.013. 23791664 10.1016/j.breast.2013.05.013

[13] Lambertini M, Anserini P, Fontana V, et al. The PREgnancy and FERtility (PREFER) study: an Italian multicenter prospective cohort study on fertility preservation and pregnancy issues in young breast cancer patients. *BMC Cancer*. 2017;17(1):346. 10.1186/s12885-017-3348-8. 28526012 10.1186/s12885-017-3348-8 PMC5437418

[14] Arecco L, Perachino M, Damassi A, et al. Burning questions in the oncofertility counseling of young breast cancer patients. *Breast Cancer (Auckl)*. 2020;14:1178223420954179. 10.1177/1178223420954179. 32952399 10.1177/1178223420954179 PMC7476336

[15] Partridge AH, Niman SM, Ruggeri M, et al. Interrupting endocrine therapy to attempt pregnancy after breast cancer. *N Engl J Med*. 2023;388(18):1645–56. 10.1056/NEJMoa2212856. 37133584 10.1056/NEJMoa2212856 PMC10358451

[16] Azim HA Jr, Santoro L, Pavlidis N, et al. Safety of pregnancy following breast cancer diagnosis: a meta-analysis of 14 studies. *Eur J Cancer*. 2011;47(1):74–83. 10.1016/j.ejca.2010.09.007. 20943370 10.1016/j.ejca.2010.09.007

[17] Lambertini M, Blondeaux E, Bruzzone M, et al. Pregnancy after breast cancer: a systematic review and meta-analysis. *J Clin Oncol*. 2021;39(29):3293–305. 10.1200/JCO.21.00535. 34197218 10.1200/JCO.21.00535

[18] Macdonald HR. Pregnancy associated breast cancer. *Breast J*. 2020;26(1):81–5. 10.1111/tbj.13714. 31943583 10.1111/tbj.13714

[19] Gerstl B, Sullivan E, Ives A, Saunders C, Wand H, Anazodo A. Pregnancy outcomes after a breast cancer diagnosis: a systematic review and meta-analysis. *Clin Breast Cancer*. 2018;18(1):e79–88. 10.1016/j.clbc.2017.06.016. 28797766 10.1016/j.clbc.2017.06.016

[20] Lambertini M, Blondeaux E, Agostinetto E, et al. Pregnancy after breast cancer in young *BRCA* carriers: an international hospital-based cohort study. *JAMA*. 2024;331(1):49–59. 10.1001/jama.2023.25463. 38059899 10.1001/jama.2023.25463 PMC10704340

